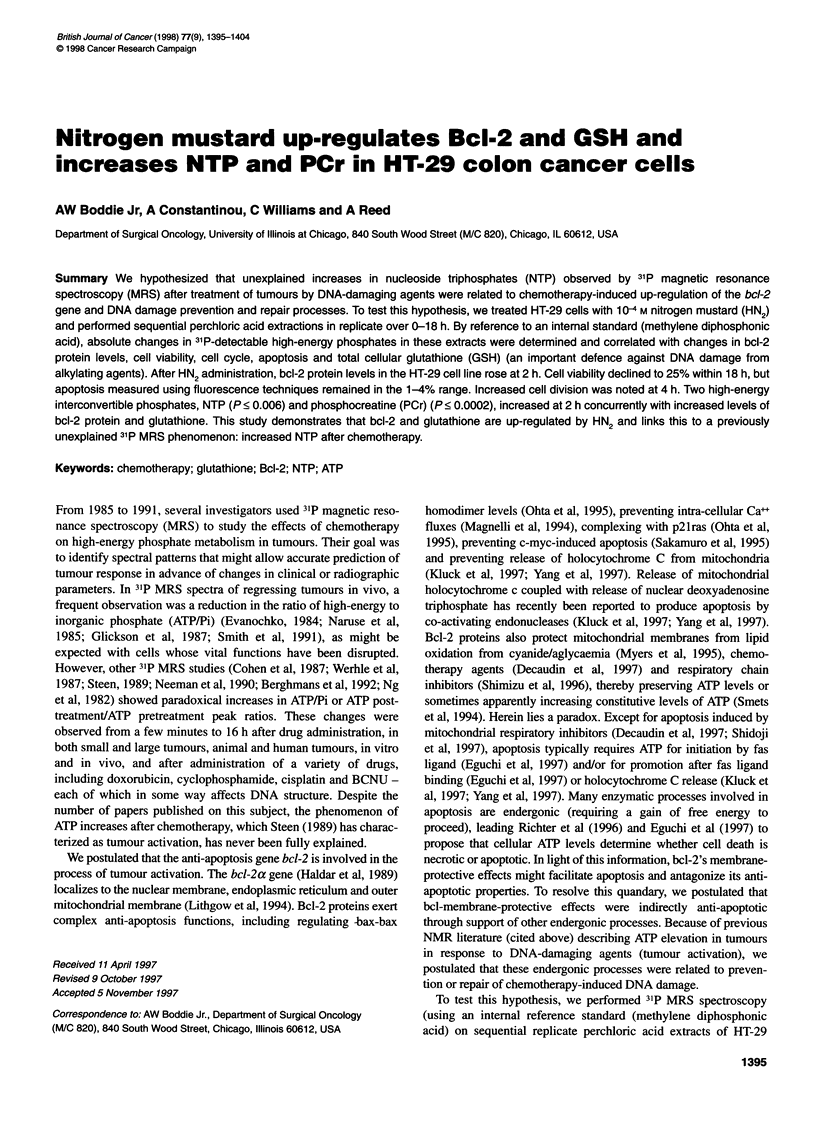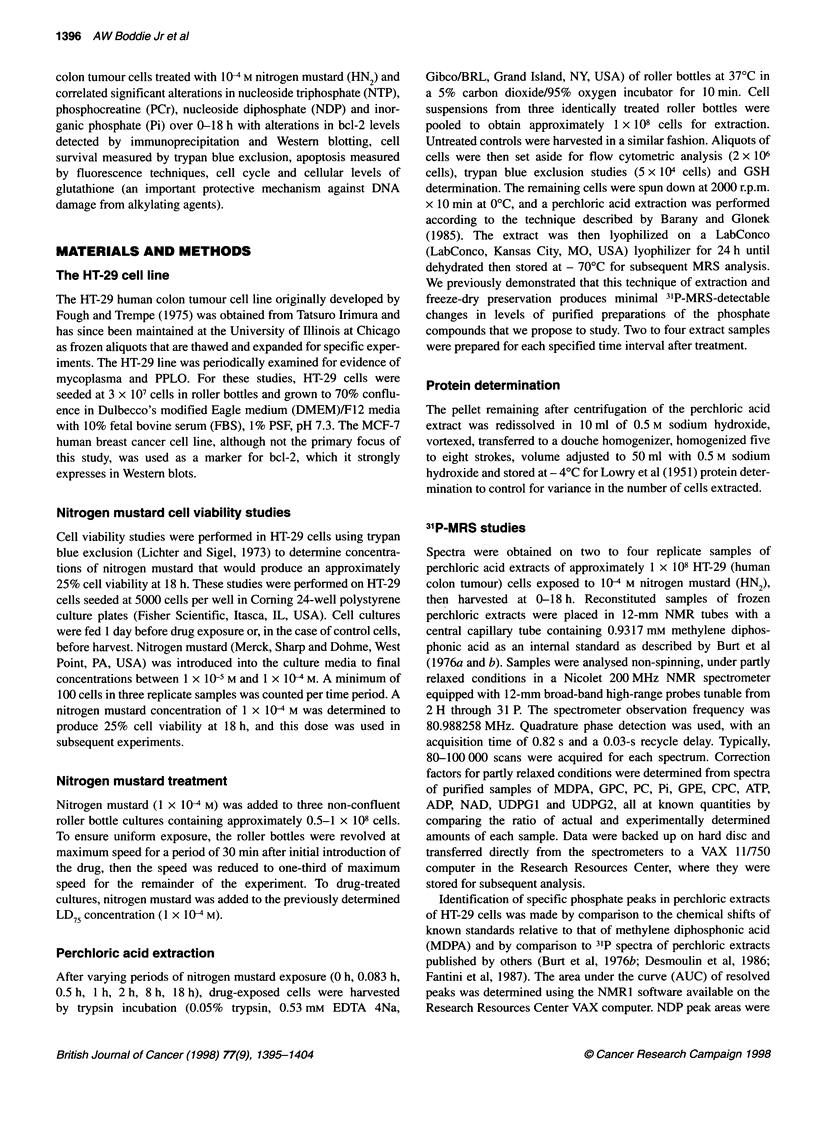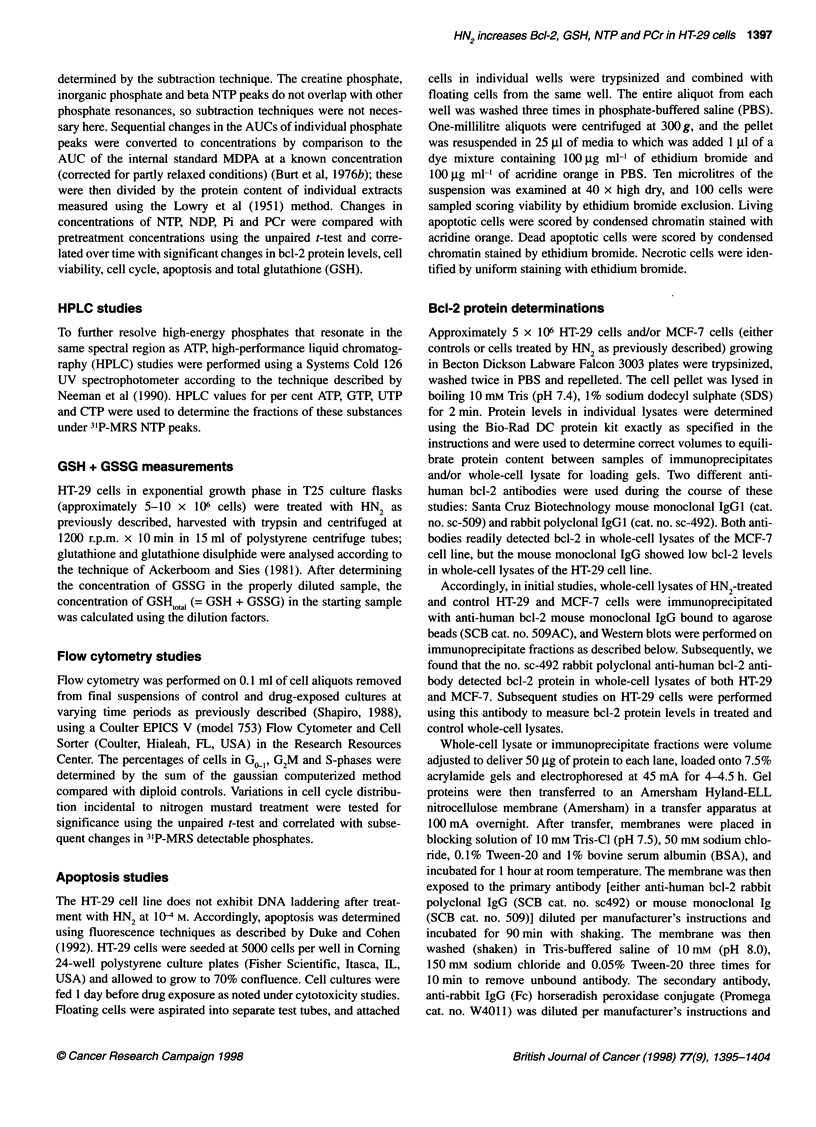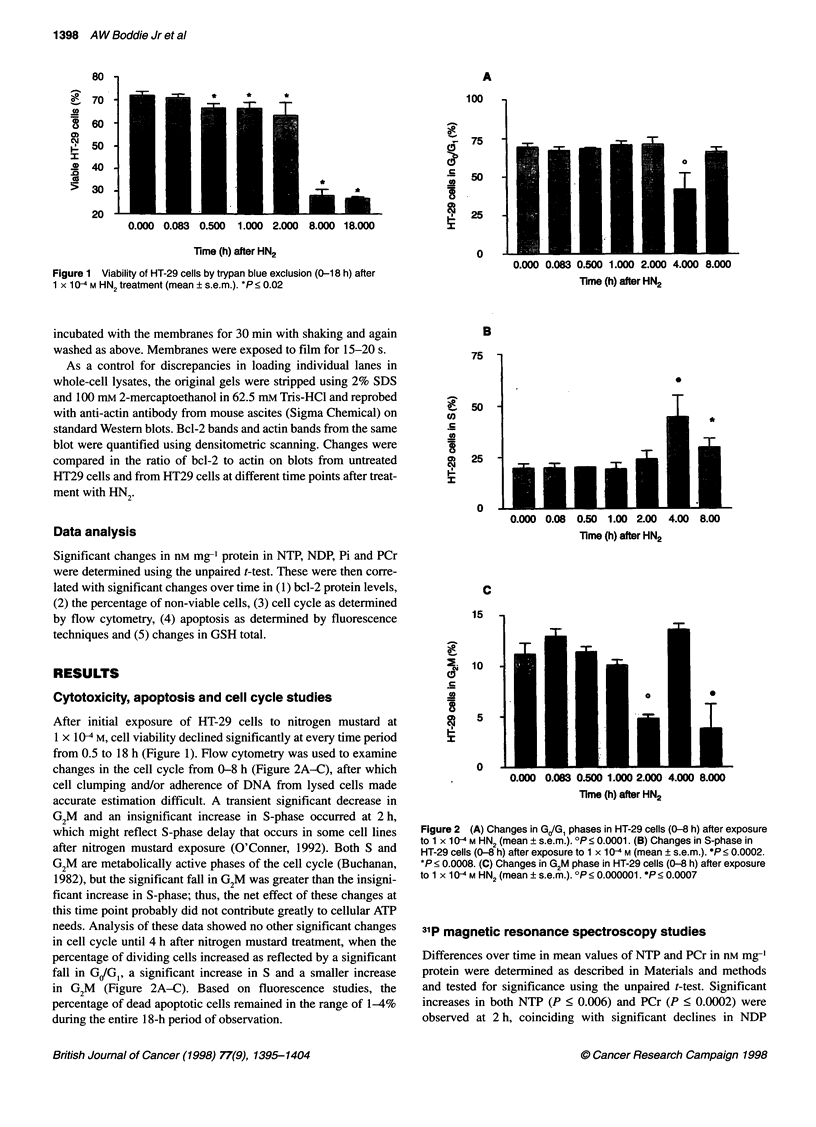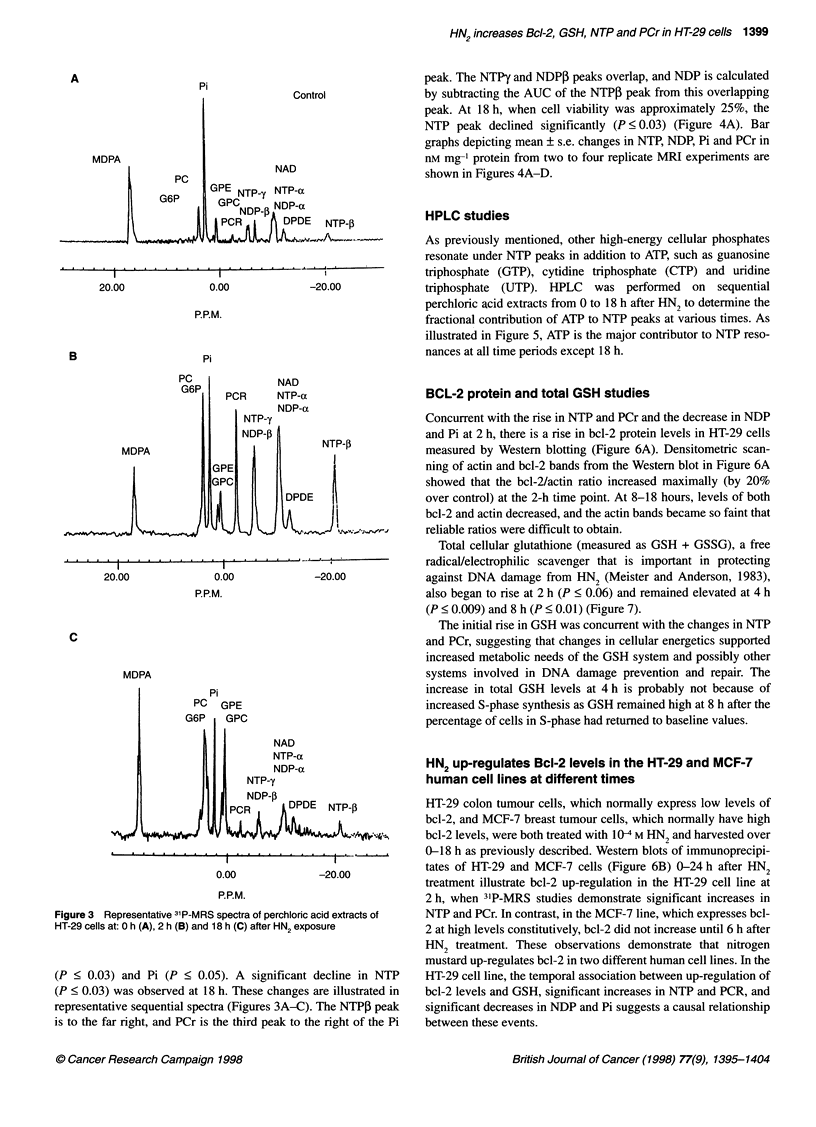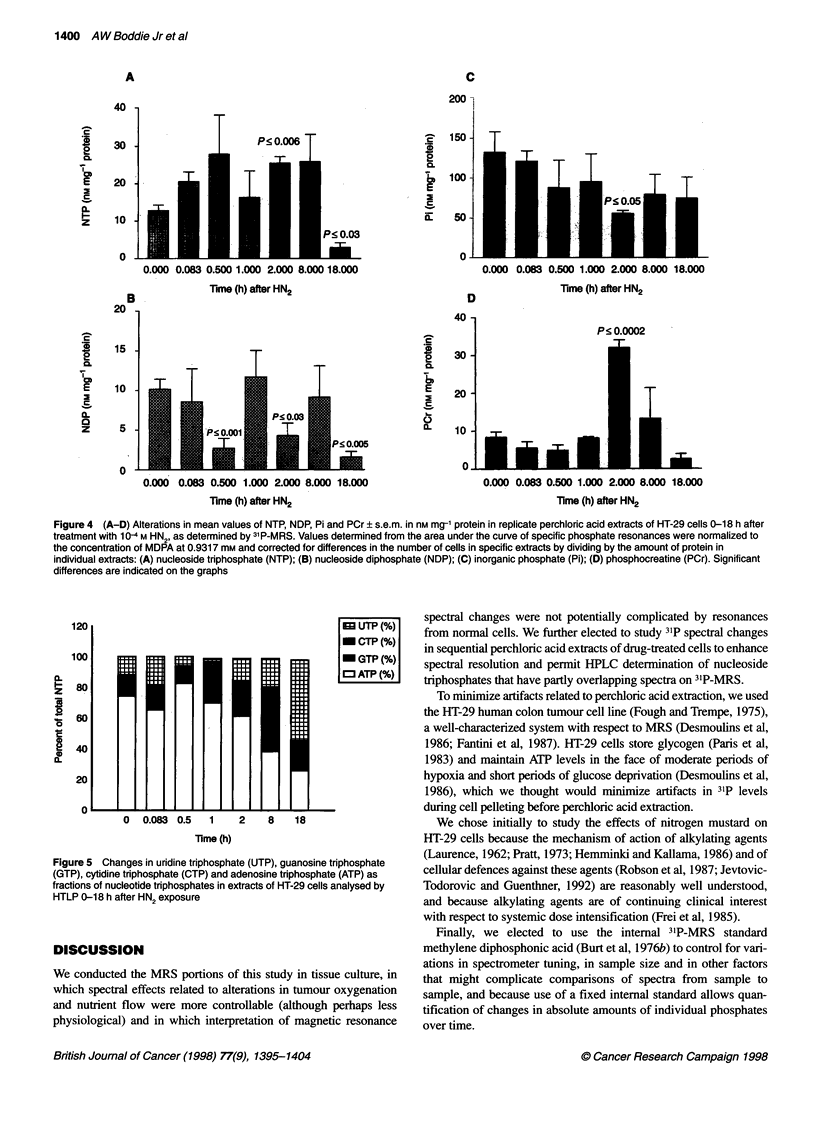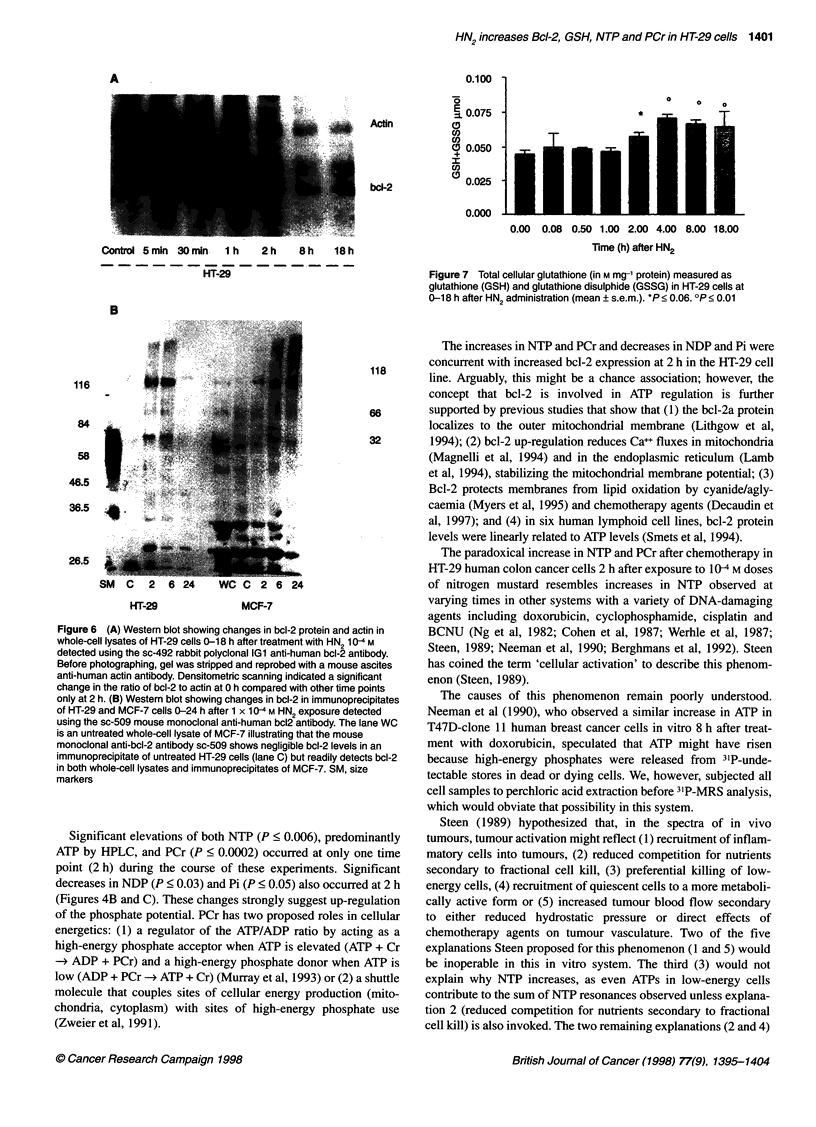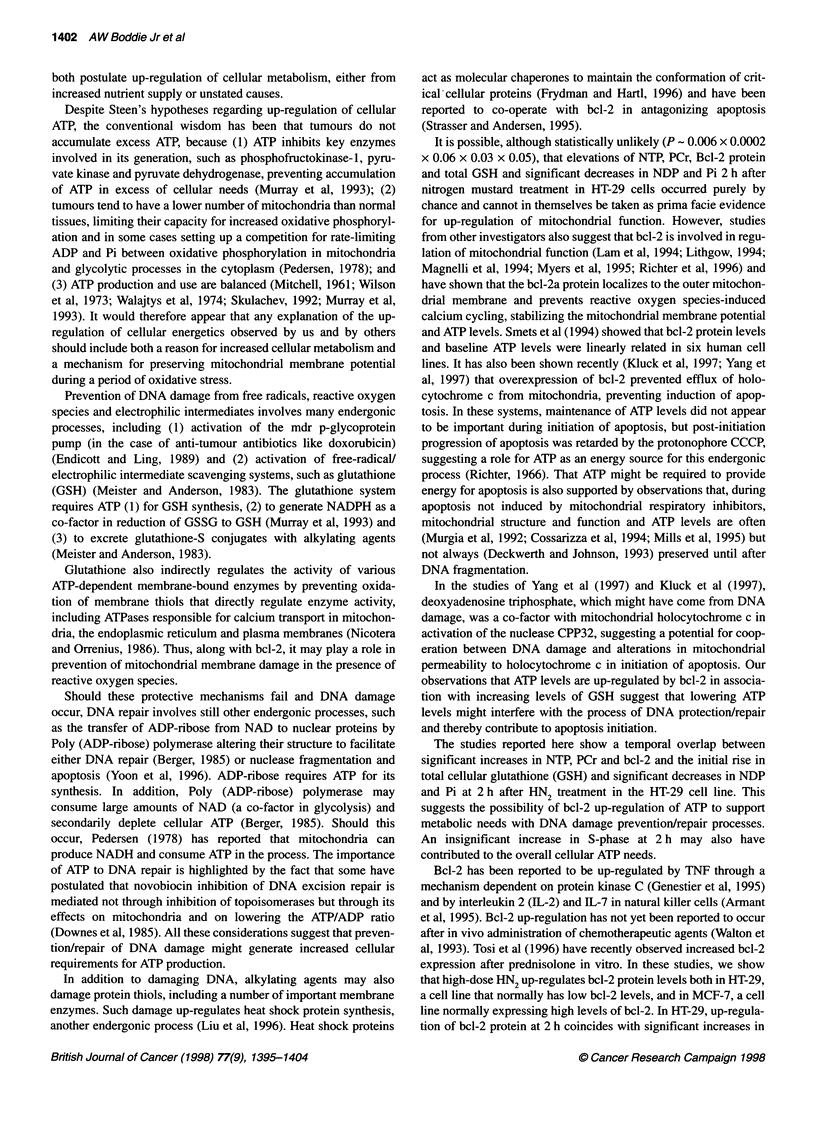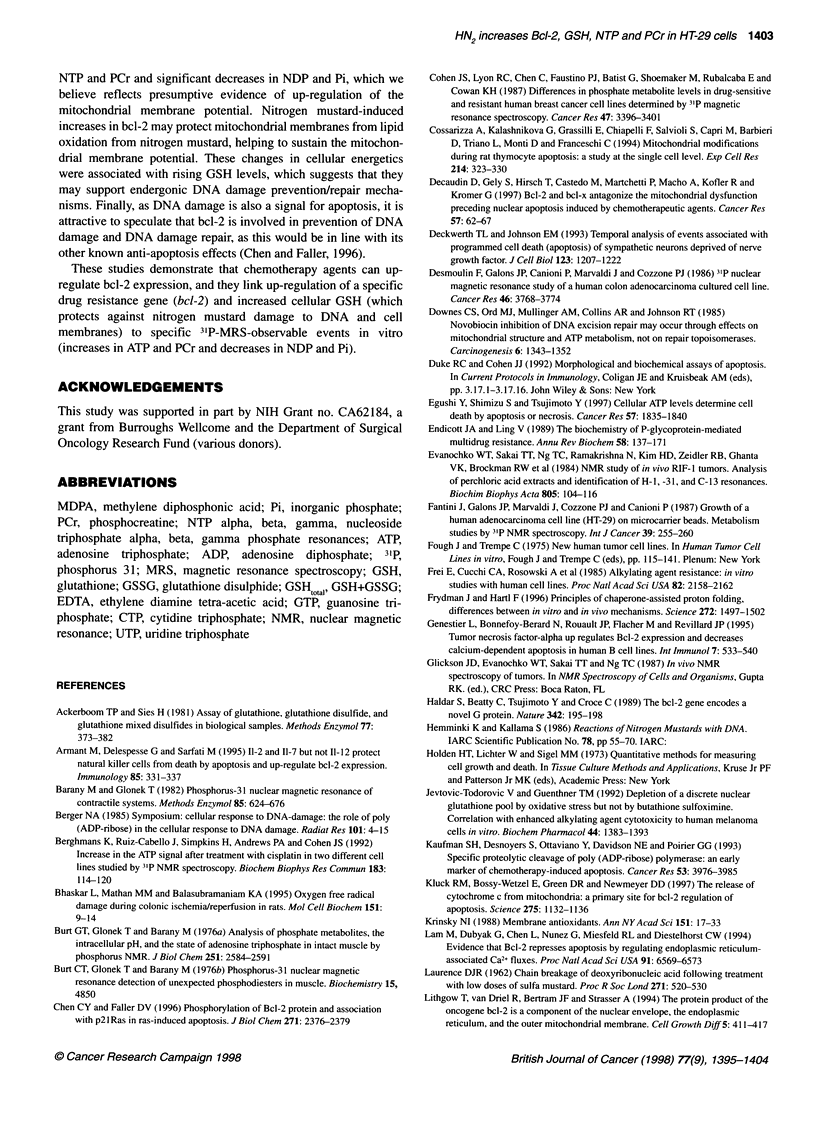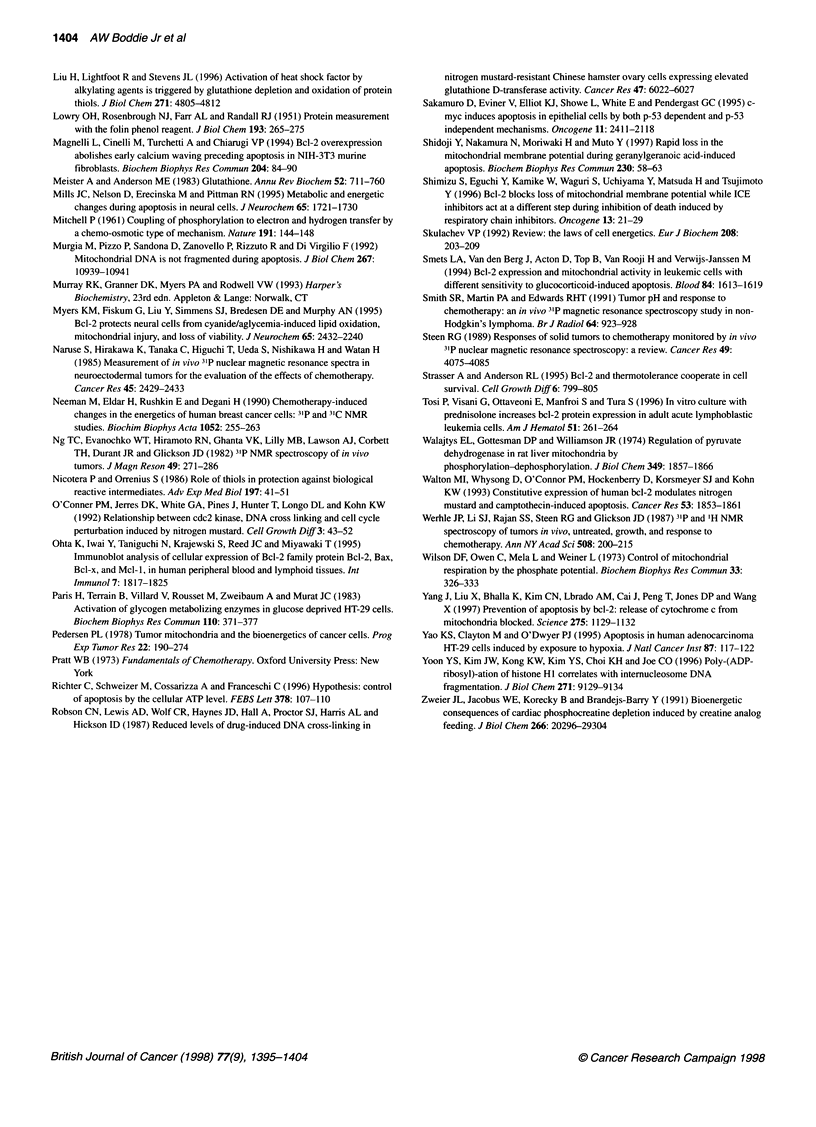# Nitrogen mustard up-regulates Bcl-2 and GSH and increases NTP and PCr in HT-29 colon cancer cells.

**DOI:** 10.1038/bjc.1998.232

**Published:** 1998-05

**Authors:** A. W. Boddie, A. Constantinou, C. Williams, A. Reed

**Affiliations:** Department of Surgical Oncology, University of Illinois at Chicago, 60612, USA.

## Abstract

**Images:**


British Journal of Cancer (1998) 77(9), 1395–1404
© 1998 Cancer Research Campaign

### Nitrogen mustard up-regulates Bcl-2 and GSH and increases NTP and PCr in HT-29 colon cancer cells

**AW Boddie Jr, A Constantinou, C Williams and A Reed**

Department of Surgical Oncology, University of Illinois at Chicago, 840 South Wood Street (M/C 820), Chicago, IL 60612, USA

**Summary** We hypothesized that unexplained increases in nucleoside triphosphates (NTP) observed by $^{31}P$ magnetic resonance spectroscopy (MRS) after treatment of tumours by DNA-damaging agents were related to chemotherapy-induced up-regulation of the *bcl-2* gene and DNA damage prevention and repair processes. To test this hypothesis, we treated HT-29 cells with $10^{-4}$ M nitrogen mustard ($HN_2$) and performed sequential perchloric acid extractions in replicate over 0–18 h. By reference to an internal standard (methylene diphosphonic acid), absolute changes in $^{31}P$-detectable high-energy phosphates in these extracts were determined and correlated with changes in bcl-2 protein levels, cell viability, cell cycle, apoptosis and total cellular glutathione (GSH) (an important defence against DNA damage from alkylating agents). After $HN_2$ administration, bcl-2 protein levels in the HT-29 cell line rose at 2 h. Cell viability declined to 25% within 18 h, but apoptosis measured using fluorescence techniques remained in the 1–4% range. Increased cell division was noted at 4 h. Two high-energy interconvertible phosphates, NTP ($P \leq 0.006$) and phosphocreatine (PCr) ($P \leq 0.0002$), increased at 2 h concurrently with increased levels of bcl-2 protein and glutathione. This study demonstrates that bcl-2 and glutathione are up-regulated by $HN_2$ and links this to a previously unexplained $^{31}P$ MRS phenomenon: increased NTP after chemotherapy.

**Keywords:** chemotherapy; glutathione; Bcl-2; NTP; ATP

From 1985 to 1991, several investigators used $^{31}P$ magnetic resonance spectroscopy (MRS) to study the effects of chemotherapy on high-energy phosphate metabolism in tumours. Their goal was to identify spectral patterns that might allow accurate prediction of tumour response in advance of changes in clinical or radiographic parameters. In $^{31}P$ MRS spectra of regressing tumours in vivo, a frequent observation was a reduction in the ratio of high-energy to inorganic phosphate (ATP/Pi) (Evanochko, 1984; Naruse et al, 1985; Glickson et al, 1987; Smith et al, 1991), as might be expected with cells whose vital functions have been disrupted. However, other $^{31}P$ MRS studies (Cohen et al, 1987; Werhle et al, 1987; Steen, 1989; Neeman et al, 1990; Berghmans et al, 1992; Ng et al, 1982) showed paradoxical increases in ATP/Pi or ATP post-treatment/ATP pretreatment peak ratios. These changes were observed from a few minutes to 16 h after drug administration, in both small and large tumours, animal and human tumours, in vitro and in vivo, and after administration of a variety of drugs, including doxorubicin, cyclophosphamide, cisplatin and BCNU – each of which in some way affects DNA structure. Despite the number of papers published on this subject, the phenomenon of ATP increases after chemotherapy, which Steen (1989) has characterized as tumour activation, has never been fully explained.

We postulated that the anti-apoptosis gene *bcl-2* is involved in the process of tumour activation. The *bcl-2α* gene (Haldar et al, 1989) localizes to the nuclear membrane, endoplasmic reticulum and outer mitochondrial membrane (Lithgow et al, 1994). Bcl-2 proteins exert complex anti-apoptosis functions, including regulating bax-bax homodimer levels (Ohta et al, 1995), preventing intra-cellular $Ca^{++}$ fluxes (Magnelli et al, 1994), complexing with p21ras (Ohta et al, 1995), preventing c-myc-induced apoptosis (Sakamuro et al, 1995) and preventing release of holocytochrome C from mitochondria (Kluck et al, 1997; Yang et al, 1997). Release of mitochondrial holocytochrome c coupled with release of nuclear deoxyadenosine triphosphate has recently been reported to produce apoptosis by co-activating endonucleases (Kluck et al, 1997; Yang et al, 1997). Bcl-2 proteins also protect mitochondrial membranes from lipid oxidation from cyanide/aglycaemia (Myers et al, 1995), chemotherapy agents (Decaudin et al, 1997) and respiratory chain inhibitors (Shimizu et al, 1996), thereby preserving ATP levels or sometimes apparently increasing constitutive levels of ATP (Smets et al, 1994). Herein lies a paradox. Except for apoptosis induced by mitochondrial respiratory inhibitors (Decaudin et al, 1997; Shidoji et al, 1997), apoptosis typically requires ATP for initiation by fas ligand (Eguchi et al, 1997) and/or for promotion after fas ligand binding (Eguchi et al, 1997) or holocytochrome C release (Kluck et al, 1997; Yang et al, 1997). Many enzymatic processes involved in apoptosis are endergonic (requiring a gain of free energy to proceed), leading Richter et al (1996) and Eguchi et al (1997) to propose that cellular ATP levels determine whether cell death is necrotic or apoptotic. In light of this information, bcl-2's membrane-protective effects might facilitate apoptosis and antagonize its anti-apoptotic properties. To resolve this quandary, we postulated that bcl-membrane-protective effects were indirectly anti-apoptotic through support of other endergonic processes. Because of previous NMR literature (cited above) describing ATP elevation in tumours in response to DNA-damaging agents (tumour activation), we postulated that these endergonic processes were related to prevention or repair of chemotherapy-induced DNA damage.

To test this hypothesis, we performed $^{31}P$ MRS spectroscopy (using an internal reference standard (methylene diphosphonic acid) on sequential replicate perchloric acid extracts of HT-29

*Received 11 April 1997*
*Revised 9 October 1997*
*Accepted 5 November 1997*

*Correspondence to:* AW Boddie Jr., Department of Surgical Oncology (M/C 820), 840 South Wood Street, Chicago, Illinois 60612, USA

colon tumour cells treated with $10^{-4}$ M nitrogen mustard ($HN_2$) and correlated significant alterations in nucleoside triphosphate (NTP), phosphocreatine (PCr), nucleoside diphosphate (NDP) and inorganic phosphate (Pi) over 0–18 h with alterations in bcl-2 levels detected by immunoprecipitation and Western blotting, cell survival measured by trypan blue exclusion, apoptosis measured by fluorescence techniques, cell cycle and cellular levels of glutathione (an important protective mechanism against DNA damage from alkylating agents).

### MATERIALS AND METHODS

### The HT-29 cell line

The HT-29 human colon tumour cell line originally developed by Fough and Trempe (1975) was obtained from Tatsuro Irimura and has since been maintained at the University of Illinois at Chicago as frozen aliquots that are thawed and expanded for specific experiments. The HT-29 line was periodically examined for evidence of mycoplasma and PPLO. For these studies, HT-29 cells were seeded at $3 \times 10^7$ cells in roller bottles and grown to 70% confluence in Dulbecco's modified Eagle medium (DMEM)/F12 media with 10% fetal bovine serum (FBS), 1% PSF, pH 7.3. The MCF-7 human breast cancer cell line, although not the primary focus of this study, was used as a marker for bcl-2, which it strongly expresses in Western blots.

### Nitrogen mustard cell viability studies

Cell viability studies were performed in HT-29 cells using trypan blue exclusion (Lichter and Sigel, 1973) to determine concentrations of nitrogen mustard that would produce an approximately 25% cell viability at 18 h. These studies were performed on HT-29 cells seeded at 5000 cells per well in Corning 24-well polystyrene culture plates (Fisher Scientific, Itasca, IL, USA). Cell cultures were fed 1 day before drug exposure or, in the case of control cells, before harvest. Nitrogen mustard (Merck, Sharp and Dohme, West Point, PA, USA) was introduced into the culture media to final concentrations between $1 \times 10^{-5}$ M and $1 \times 10^{-4}$ M. A minimum of 100 cells in three replicate samples was counted per time period. A nitrogen mustard concentration of $1 \times 10^{-4}$ M was determined to produce 25% cell viability at 18 h, and this dose was used in subsequent experiments.

### Nitrogen mustard treatment

Nitrogen mustard ($1 \times 10^{-4}$ M) was added to three non-confluent roller bottle cultures containing approximately $0.5–1 \times 10^8$ cells. To ensure uniform exposure, the roller bottles were revolved at maximum speed for a period of 30 min after initial introduction of the drug, then the speed was reduced to one-third of maximum speed for the remainder of the experiment. To drug-treated cultures, nitrogen mustard was added to the previously determined $LD_{75}$ concentration ($1 \times 10^{-4}$ M).

### Perchloric acid extraction

After varying periods of nitrogen mustard exposure (0 h, 0.083 h, 0.5 h, 1 h, 2 h, 8 h, 18 h), drug-exposed cells were harvested by trypsin incubation (0.05% trypsin, 0.53 mM EDTA 4Na, Gibco/BRL, Grand Island, NY, USA) of roller bottles at 37°C in a 5% carbon dioxide/95% oxygen incubator for 10 min. Cell suspensions from three identically treated roller bottles were pooled to obtain approximately $1 \times 10^8$ cells for extraction. Untreated controls were harvested in a similar fashion. Aliquots of cells were then set aside for flow cytometric analysis ($2 \times 10^6$ cells), trypan blue exclusion studies ($5 \times 10^4$ cells) and GSH determination. The remaining cells were spun down at 2000 r.p.m. × 10 min at 0°C, and a perchloric acid extraction was performed according to the technique described by Barany and Glonek (1985). The extract was then lyophilized on a LabConco (LabConco, Kansas City, MO, USA) lyophilizer for 24 h until dehydrated then stored at – 70°C for subsequent MRS analysis. We previously demonstrated that this technique of extraction and freeze-dry preservation produces minimal $^{31}$P-MRS-detectable changes in levels of purified preparations of the phosphate compounds that we propose to study. Two to four extract samples were prepared for each specified time interval after treatment.

### Protein determination

The pellet remaining after centrifugation of the perchloric acid extract was redissolved in 10 ml of 0.5 M sodium hydroxide, vortexed, transferred to a douche homogenizer, homogenized five to eight strokes, volume adjusted to 50 ml with 0.5 M sodium hydroxide and stored at – 4°C for Lowry et al (1951) protein determination to control for variance in the number of cells extracted.

### $^{31}$P-MRS studies

Spectra were obtained on two to four replicate samples of perchloric acid extracts of approximately $1 \times 10^8$ HT-29 (human colon tumour) cells exposed to $10^{-4}$ M nitrogen mustard ($HN_2$), then harvested at 0–18 h. Reconstituted samples of frozen perchloric extracts were placed in 12-mm NMR tubes with a central capillary tube containing 0.9317 mM methylene diphosphonic acid as an internal standard as described by Burt et al (1976*a* and *b*). Samples were analysed non-spinning, under partly relaxed conditions in a Nicolet 200 MHz NMR spectrometer equipped with 12-mm broad-band high-range probes tunable from 2 H through 31 P. The spectrometer observation frequency was 80.988258 MHz. Quadrature phase detection was used, with an acquisition time of 0.82 s and a 0.03-s recycle delay. Typically, 80–100 000 scans were acquired for each spectrum. Correction factors for partly relaxed conditions were determined from spectra of purified samples of MDPA, GPC, PC, Pi, GPE, CPC, ATP, ADP, NAD, UDPG1 and UDPG2, all at known quantities by comparing the ratio of actual and experimentally determined amounts of each sample. Data were backed up on hard disc and transferred directly from the spectrometers to a VAX 11/750 computer in the Research Resources Center, where they were stored for subsequent analysis.

Identification of specific phosphate peaks in perchloric extracts of HT-29 cells was made by comparison to the chemical shifts of known standards relative to that of methylene diphosphonic acid (MDPA) and by comparison to $^{31}$P spectra of perchloric extracts published by others (Burt et al, 1976*b*; Desmoulin et al, 1986; Fantini et al, 1987). The area under the curve (AUC) of resolved peaks was determined using the NMR1 software available on the Research Resources Center VAX computer. NDP peak areas were

© Cancer Research Campaign 1998

determined by the subtraction technique. The creatine phosphate, inorganic phosphate and beta NTP peaks do not overlap with other phosphate resonances, so subtraction techniques were not necessary here. Sequential changes in the AUCs of individual phosphate peaks were converted to concentrations by comparison to the AUC of the internal standard MDPA at a known concentration (corrected for partly relaxed conditions) (Burt et al, 1976*b*); these were then divided by the protein content of individual extracts measured using the Lowry et al (1951) method. Changes in concentrations of NTP, NDP, Pi and PCr were compared with pretreatment concentrations using the unpaired *t*-test and correlated over time with significant changes in bcl-2 protein levels, cell viability, cell cycle, apoptosis and total glutathione (GSH).

### HPLC studies

To further resolve high-energy phosphates that resonate in the same spectral region as ATP, high-performance liquid chromatography (HPLC) studies were performed using a Systems Cold 126 UV spectrophotometer according to the technique described by Neeman et al (1990). HPLC values for per cent ATP, GTP, UTP and CTP were used to determine the fractions of these substances under $^{31}$P-MRS NTP peaks.

### GSH + GSSG measurements

HT-29 cells in exponential growth phase in T25 culture flasks (approximately 5–10 × $10^6$ cells) were treated with $HN_2$ as previously described, harvested with trypsin and centrifuged at 1200 r.p.m. × 10 min in 15 ml of polystyrene centrifuge tubes; glutathione and glutathione disulphide were analysed according to the technique of Ackerboom and Sies (1981). After determining the concentration of GSSG in the properly diluted sample, the concentration of $GSH_{total}$ (= GSH + GSSG) in the starting sample was calculated using the dilution factors.

### Flow cytometry studies

Flow cytometry was performed on 0.1 ml of cell aliquots removed from final suspensions of control and drug-exposed cultures at varying time periods as previously described (Shapiro, 1988), using a Coulter EPICS V (model 753) Flow Cytometer and Cell Sorter (Coulter, Hialeah, FL, USA) in the Research Resources Center. The percentages of cells in $G_{0-1}$, $G_2M$ and S-phases were determined by the sum of the gaussian computerized method compared with diploid controls. Variations in cell cycle distribution incidental to nitrogen mustard treatment were tested for significance using the unpaired *t*-test and correlated with subsequent changes in $^{31}$P-MRS detectable phosphates.

### Apoptosis studies

The HT-29 cell line does not exhibit DNA laddering after treatment with $HN_2$ at $10^{-4}$ M. Accordingly, apoptosis was determined using fluorescence techniques as described by Duke and Cohen (1992). HT-29 cells were seeded at 5000 cells per well in Corning 24-well polystyrene culture plates (Fisher Scientific, Itasca, IL, USA) and allowed to grow to 70% confluence. Cell cultures were fed 1 day before drug exposure as noted under cytotoxicity studies. Floating cells were aspirated into separate test tubes, and attached cells in individual wells were trypsinized and combined with floating cells from the same well. The entire aliquot from each well was washed three times in phosphate-buffered saline (PBS). One-millilitre aliquots were centrifuged at 300 ***g***, and the pellet was resuspended in 25 µl of media to which was added 1 µl of a dye mixture containing 100 µg $ml^{-1}$ of ethidium bromide and 100 µg $ml^{-1}$ of acridine orange in PBS. Ten microlitres of the suspension was examined at 40 × high dry, and 100 cells were sampled scoring viability by ethidium bromide exclusion. Living apoptotic cells were scored by condensed chromatin stained with acridine orange. Dead apoptotic cells were scored by condensed chromatin stained by ethidium bromide. Necrotic cells were identified by uniform staining with ethidium bromide.

### Bcl-2 protein determinations

Approximately 5 × $10^6$ HT-29 cells and/or MCF-7 cells (either controls or cells treated by $HN_2$ as previously described) growing in Becton Dickson Labware Falcon 3003 plates were trypsinized, washed twice in PBS and repelleted. The cell pellet was lysed in boiling 10 mM Tris (pH 7.4), 1% sodium dodecyl sulphate (SDS) for 2 min. Protein levels in individual lysates were determined using the Bio-Rad DC protein kit exactly as specified in the instructions and were used to determine correct volumes to equilibrate protein content between samples of immunoprecipitates and/or whole-cell lysate for loading gels. Two different anti-human bcl-2 antibodies were used during the course of these studies: Santa Cruz Biotechnology mouse monoclonal IgG1 (cat. no. sc-509) and rabbit polyclonal IgG1 (cat. no. sc-492). Both antibodies readily detected bcl-2 in whole-cell lysates of the MCF-7 cell line, but the mouse monoclonal IgG showed low bcl-2 levels in whole-cell lysates of the HT-29 cell line.

Accordingly, in initial studies, whole-cell lysates of $HN_2$-treated and control HT-29 and MCF-7 cells were immunoprecipitated with anti-human bcl-2 mouse monoclonal IgG bound to agarose beads (SCB cat. no. 509AC), and Western blots were performed on immunoprecipitate fractions as described below. Subsequently, we found that the no. sc-492 rabbit polyclonal anti-human bcl-2 antibody detected bcl-2 protein in whole-cell lysates of both HT-29 and MCF-7. Subsequent studies on HT-29 cells were performed using this antibody to measure bcl-2 protein levels in treated and control whole-cell lysates.

Whole-cell lysate or immunoprecipitate fractions were volume adjusted to deliver 50 µg of protein to each lane, loaded onto 7.5% acrylamide gels and electrophoresed at 45 mA for 4–4.5 h. Gel proteins were then transferred to an Amersham Hyland-ELL nitrocellulose membrane (Amersham) in a transfer apparatus at 100 mA overnight. After transfer, membranes were placed in blocking solution of 10 mM Tris-Cl (pH 7.5), 50 mM sodium chloride, 0.1% Tween-20 and 1% bovine serum albumin (BSA), and incubated for 1 hour at room temperature. The membrane was then exposed to the primary antibody [either anti-human bcl-2 rabbit polyclonal IgG (SCB cat. no. sc492) or mouse monoclonal Ig (SCB cat. no. 509)] diluted per manufacturer's instructions and incubated for 90 min with shaking. The membrane was then washed (shaken) in Tris-buffered saline of 10 mM (pH 8.0), 150 mM sodium chloride and 0.05% Tween-20 three times for 10 min to remove unbound antibody. The secondary antibody, anti-rabbit IgG (Fc) horseradish peroxidase conjugate (Promega cat. no. W4011) was diluted per manufacturer's instructions and

© Cancer Research Campaign 1998

**Figure 1** Viability of HT-29 cells by trypan blue exclusion (0–18 h) after 1 × $10^{-4}$ M $HN_2$ treatment (mean ± s.e.m.). *$P \leq 0.02$

incubated with the membranes for 30 min with shaking and again washed as above. Membranes were exposed to film for 15–20 s.

As a control for discrepancies in loading individual lanes in whole-cell lysates, the original gels were stripped using 2% SDS and 100 mM 2-mercaptoethanol in 62.5 mM Tris-HCl and reprobed with anti-actin antibody from mouse ascites (Sigma Chemical) on standard Western blots. Bcl-2 bands and actin bands from the same blot were quantified using densitometric scanning. Changes were compared in the ratio of bcl-2 to actin on blots from untreated HT29 cells and from HT29 cells at different time points after treatment with $HN_2$.

### Data analysis

Significant changes in nM $mg^{-1}$ protein in NTP, NDP, Pi and PCr were determined using the unpaired *t*-test. These were then correlated with significant changes over time in (1) bcl-2 protein levels, (2) the percentage of non-viable cells, (3) cell cycle as determined by flow cytometry, (4) apoptosis as determined by fluorescence techniques and (5) changes in GSH total.

### RESULTS

### Cytotoxicity, apoptosis and cell cycle studies

After initial exposure of HT-29 cells to nitrogen mustard at 1 × $10^{-4}$ M, cell viability declined significantly at every time period from 0.5 to 18 h (Figure 1). Flow cytometry was used to examine changes in the cell cycle from 0–8 h (Figure 2A–C), after which cell clumping and/or adherence of DNA from lysed cells made accurate estimation difficult. A transient significant decrease in $G_2M$ and an insignificant increase in S-phase occurred at 2 h, which might reflect S-phase delay that occurs in some cell lines after nitrogen mustard exposure (O'Conner, 1992). Both S and $G_2M$ are metabolically active phases of the cell cycle (Buchanan, 1982), but the significant fall in $G_2M$ was greater than the insignificant increase in S-phase; thus, the net effect of these changes at this time point probably did not contribute greatly to cellular ATP needs. Analysis of these data showed no other significant changes in cell cycle until 4 h after nitrogen mustard treatment, when the percentage of dividing cells increased as reflected by a significant fall in $G_0/G_1$, a significant increase in S and a smaller increase in $G_2M$ (Figure 2A–C). Based on fluorescence studies, the percentage of dead apoptotic cells remained in the range of 1–4% during the entire 18-h period of observation.

**Figure 2** (**A**) Changes in $G_0/G_1$ phases in HT-29 cells (0–8 h) after exposure to 1 × $10^{-4}$ M $HN_2$ (mean ± s.e.m.). °$P \leq 0.0001$. (**B**) Changes in S-phase in HT-29 cells (0–8 h) after exposure to 1 × $10^{-4}$ M (mean ± s.e.m.). •$P \leq 0.0002$. *$P \leq 0.0008$. (**C**) Changes in $G_2M$ phase in HT-29 cells (0–8 h) after exposure to 1 × $10^{-4}$ M $HN_2$ (mean ± s.e.m.). °$P \leq 0.000001$. •$P \leq 0.0007$

### $^{31}P$ magnetic resonance spectroscopy studies

Differences over time in mean values of NTP and PCr in nM $mg^{-1}$ protein were determined as described in Materials and methods and tested for significance using the unpaired *t*-test. Significant increases in both NTP ($P \leq 0.006$) and PCr ($P \leq 0.0002$) were observed at 2 h, coinciding with significant declines in NDP

© Cancer Research Campaign 1998

**Figure 3** Representative $^{31}$P-MRS spectra of perchloric acid extracts of HT-29 cells at: 0 h (**A**), 2 h (**B**) and 18 h (**C**) after $HN_2$ exposure

($P \leq 0.03$) and Pi ($P \leq 0.05$). A significant decline in NTP ($P \leq 0.03$) was observed at 18 h. These changes are illustrated in representative sequential spectra (Figures 3A–C). The NTPβ peak is to the far right, and PCr is the third peak to the right of the Pi peak. The NTPγ and NDPβ peaks overlap, and NDP is calculated by subtracting the AUC of the NTPβ peak from this overlapping peak. At 18 h, when cell viability was approximately 25%, the NTP peak declined significantly ($P \leq 0.03$) (Figure 4A). Bar graphs depicting mean ± s.e. changes in NTP, NDP, Pi and PCr in nM $mg^{-1}$ protein from two to four replicate MRI experiments are shown in Figures 4A–D.

### HPLC studies

As previously mentioned, other high-energy cellular phosphates resonate under NTP peaks in addition to ATP, such as guanosine triphosphate (GTP), cytidine triphosphate (CTP) and uridine triphosphate (UTP). HPLC was performed on sequential perchloric acid extracts from 0 to 18 h after $HN_2$ to determine the fractional contribution of ATP to NTP peaks at various times. As illustrated in Figure 5, ATP is the major contributor to NTP resonances at all time periods except 18 h.

### BCL-2 protein and total GSH studies

Concurrent with the rise in NTP and PCr and the decrease in NDP and Pi at 2 h, there is a rise in bcl-2 protein levels in HT-29 cells measured by Western blotting (Figure 6A). Densitometric scanning of actin and bcl-2 bands from the Western blot in Figure 6A showed that the bcl-2/actin ratio increased maximally (by 20% over control) at the 2-h time point. At 8–18 hours, levels of both bcl-2 and actin decreased, and the actin bands became so faint that reliable ratios were difficult to obtain.

Total cellular glutathione (measured as GSH + GSSG), a free radical/electrophilic scavenger that is important in protecting against DNA damage from $HN_2$ (Meister and Anderson, 1983), also began to rise at 2 h ($P \leq 0.06$) and remained elevated at 4 h ($P \leq 0.009$) and 8 h ($P \leq 0.01$) (Figure 7).

The initial rise in GSH was concurrent with the changes in NTP and PCr, suggesting that changes in cellular energetics supported increased metabolic needs of the GSH system and possibly other systems involved in DNA damage prevention and repair. The increase in total GSH levels at 4 h is probably not because of increased S-phase synthesis as GSH remained high at 8 h after the percentage of cells in S-phase had returned to baseline values.

### $HN_2$ up-regulates Bcl-2 levels in the HT-29 and MCF-7 human cell lines at different times

HT-29 colon tumour cells, which normally express low levels of bcl-2, and MCF-7 breast tumour cells, which normally have high bcl-2 levels, were both treated with $10^{-4}$ M $HN_2$ and harvested over 0–18 h as previously described. Western blots of immunoprecipitates of HT-29 and MCF-7 cells (Figure 6B) 0–24 h after $HN_2$ treatment illustrate bcl-2 up-regulation in the HT-29 cell line at 2 h, when $^{31}$P-MRS studies demonstrate significant increases in NTP and PCr. In contrast, in the MCF-7 line, which expresses bcl-2 at high levels constitutively, bcl-2 did not increase until 6 h after $HN_2$ treatment. These observations demonstrate that nitrogen mustard up-regulates bcl-2 in two different human cell lines. In the HT-29 cell line, the temporal association between up-regulation of bcl-2 levels and GSH, significant increases in NTP and PCR, and significant decreases in NDP and Pi suggests a causal relationship between these events.

**Figure 4** (**A–D**) Alterations in mean values of NTP, NDP, Pi and PCr ± s.e.m. in nM mg⁻¹ protein in replicate perchloric acid extracts of HT-29 cells 0–18 h after treatment with $10^{-4}$ M $HN_2$, as determined by $^{31}$P-MRS. Values determined from the area under the curve of specific phosphate resonances were normalized to the concentration of MDPA at 0.9317 mM and corrected for differences in the number of cells in specific extracts by dividing by the amount of protein in individual extracts: (**A**) nucleoside triphosphate (NTP); (**B**) nucleoside diphosphate (NDP); (**C**) inorganic phosphate (Pi); (**D**) phosphocreatine (PCr). Significant differences are indicated on the graphs

**Figure 5** Changes in uridine triphosphate (UTP), guanosine triphosphate (GTP), cytidine triphosphate (CTP) and adenosine triphosphate (ATP) as fractions of nucleotide triphosphates in extracts of HT-29 cells analysed by HTLP 0–18 h after $HN_2$ exposure

### DISCUSSION

We conducted the MRS portions of this study in tissue culture, in which spectral effects related to alterations in tumour oxygenation and nutrient flow were more controllable (although perhaps less physiological) and in which interpretation of magnetic resonance spectral changes were not potentially complicated by resonances from normal cells. We further elected to study $^{31}$P spectral changes in sequential perchloric acid extracts of drug-treated cells to enhance spectral resolution and permit HPLC determination of nucleoside triphosphates that have partly overlapping spectra on $^{31}$P-MRS.

To minimize artifacts related to perchloric acid extraction, we used the HT-29 human colon tumour cell line (Fough and Trempe, 1975), a well-characterized system with respect to MRS (Desmoulins et al, 1986; Fantini et al, 1987). HT-29 cells store glycogen (Paris et al, 1983) and maintain ATP levels in the face of moderate periods of hypoxia and short periods of glucose deprivation (Desmoulins et al, 1986), which we thought would minimize artifacts in $^{31}$P levels during cell pelleting before perchloric acid extraction.

We chose initially to study the effects of nitrogen mustard on HT-29 cells because the mechanism of action of alkylating agents (Laurence, 1962; Pratt, 1973; Hemminki and Kallama, 1986) and of cellular defences against these agents (Robson et al, 1987; Jevtovic-Todorovic and Guenthner, 1992) are reasonably well understood, and because alkylating agents are of continuing clinical interest with respect to systemic dose intensification (Frei et al, 1985).

Finally, we elected to use the internal $^{31}$P-MRS standard methylene diphosphonic acid (Burt et al, 1976*b*) to control for variations in spectrometer tuning, in sample size and in other factors that might complicate comparisons of spectra from sample to sample, and because use of a fixed internal standard allows quantification of changes in absolute amounts of individual phosphates over time.

© Cancer Research Campaign 1998

**Figure 6** (**A**) Western blot showing changes in bcl-2 protein and actin in whole-cell lysates of HT-29 cells 0–18 h after treatment with $HN_2$ $10^{-4}$ M detected using the sc-492 rabbit polyclonal IG1 anti-human bcl-2 antibody. Before photographing, gel was stripped and reprobed with a mouse ascites anti-human actin antibody. Densitometric scanning indicated a significant change in the ratio of bcl-2 to actin at 0 h compared with other time points only at 2 h. (**B**) Western blot showing changes in bcl-2 in immunoprecipitates of HT-29 and MCF-7 cells 0–24 h after $1 \times 10^{-4}$ M $HN_2$ exposure detected using the sc-509 mouse monoclonal anti-human bcl2 antibody. The lane WC is an untreated whole-cell lysate of MCF-7 illustrating that the mouse monoclonal anti-bcl-2 antibody sc-509 shows negligible bcl-2 levels in an immunoprecipitate of untreated HT-29 cells (lane C) but readily detects bcl-2 in both whole-cell lysates and immunoprecipitates of MCF-7. SM, size markers

**Figure 7** Total cellular glutathione (in M $mg^{-1}$ protein) measured as glutathione (GSH) and glutathione disulphide (GSSG) in HT-29 cells at 0–18 h after $HN_2$ administration (mean ± s.e.m.). $^*P \leq 0.06$. $^\circ P \leq 0.01$

Significant elevations of both NTP ($P \leq 0.006$), predominantly ATP by HPLC, and PCr ($P \leq 0.0002$) occurred at only one time point (2 h) during the course of these experiments. Significant decreases in NDP ($P \leq 0.03$) and Pi ($P \leq 0.05$) also occurred at 2 h (Figures 4B and C). These changes strongly suggest up-regulation of the phosphate potential. PCr has two proposed roles in cellular energetics: (1) a regulator of the ATP/ADP ratio by acting as a high-energy phosphate acceptor when ATP is elevated (ATP + Cr $\rightarrow$ ADP + PCr) and a high-energy phosphate donor when ATP is low (ADP + PCr $\rightarrow$ ATP + Cr) (Murray et al, 1993) or (2) a shuttle molecule that couples sites of cellular energy production (mitochondria, cytoplasm) with sites of high-energy phosphate use (Zweier et al, 1991).

The increases in NTP and PCr and decreases in NDP and Pi were concurrent with increased bcl-2 expression at 2 h in the HT-29 cell line. Arguably, this might be a chance association; however, the concept that bcl-2 is involved in ATP regulation is further supported by previous studies that show that (1) the bcl-2a protein localizes to the outer mitochondrial membrane (Lithgow et al, 1994); (2) bcl-2 up-regulation reduces $Ca^{++}$ fluxes in mitochondria (Magnelli et al, 1994) and in the endoplasmic reticulum (Lamb et al, 1994), stabilizing the mitochondrial membrane potential; (3) Bcl-2 protects membranes from lipid oxidation by cyanide/aglycaemia (Myers et al, 1995) and chemotherapy agents (Decaudin et al, 1997); and (4) in six human lymphoid cell lines, bcl-2 protein levels were linearly related to ATP levels (Smets et al, 1994).

The paradoxical increase in NTP and PCr after chemotherapy in HT-29 human colon cancer cells 2 h after exposure to $10^{-4}$ M doses of nitrogen mustard resembles increases in NTP observed at varying times in other systems with a variety of DNA-damaging agents including doxorubicin, cyclophosphamide, cisplatin and BCNU (Ng et al, 1982; Cohen et al, 1987; Werhle et al, 1987; Steen, 1989; Neeman et al, 1990; Berghmans et al, 1992). Steen has coined the term 'cellular activation' to describe this phenomenon (Steen, 1989).

The causes of this phenomenon remain poorly understood. Neeman et al (1990), who observed a similar increase in ATP in T47D-clone 11 human breast cancer cells in vitro 8 h after treatment with doxorubicin, speculated that ATP might have risen because high-energy phosphates were released from $^{31}$P-undetectable stores in dead or dying cells. We, however, subjected all cell samples to perchloric acid extraction before $^{31}$P-MRS analysis, which would obviate that possibility in this system.

Steen (1989) hypothesized that, in the spectra of in vivo tumours, tumour activation might reflect (1) recruitment of inflammatory cells into tumours, (2) reduced competition for nutrients secondary to fractional cell kill, (3) preferential killing of low-energy cells, (4) recruitment of quiescent cells to a more metabolically active form or (5) increased tumour blood flow secondary to either reduced hydrostatic pressure or direct effects of chemotherapy agents on tumour vasculature. Two of the five explanations Steen proposed for this phenomenon (1 and 5) would be inoperable in this in vitro system. The third (3) would not explain why NTP increases, as even ATPs in low-energy cells contribute to the sum of NTP resonances observed unless explanation 2 (reduced competition for nutrients secondary to fractional cell kill) is also invoked. The two remaining explanations (2 and 4)

© Cancer Research Campaign 1998

both postulate up-regulation of cellular metabolism, either from increased nutrient supply or unstated causes.

Despite Steen's hypotheses regarding up-regulation of cellular ATP, the conventional wisdom has been that tumours do not accumulate excess ATP, because (1) ATP inhibits key enzymes involved in its generation, such as phosphofructokinase-1, pyruvate kinase and pyruvate dehydrogenase, preventing accumulation of ATP in excess of cellular needs (Murray et al, 1993); (2) tumours tend to have a lower number of mitochondria than normal tissues, limiting their capacity for increased oxidative phosphorylation and in some cases setting up a competition for rate-limiting ADP and Pi between oxidative phosphorylation in mitochondria and glycolytic processes in the cytoplasm (Pedersen, 1978); and (3) ATP production and use are balanced (Mitchell, 1961; Wilson et al, 1973; Walajtys et al, 1974; Skulachev, 1992; Murray et al, 1993). It would therefore appear that any explanation of the up-regulation of cellular energetics observed by us and by others should include both a reason for increased cellular metabolism and a mechanism for preserving mitochondrial membrane potential during a period of oxidative stress.

Prevention of DNA damage from free radicals, reactive oxygen species and electrophilic intermediates involves many endergonic processes, including (1) activation of the mdr p-glycoprotein pump (in the case of anti-tumour antibiotics like doxorubicin) (Endicott and Ling, 1989) and (2) activation of free-radical/electrophilic intermediate scavenging systems, such as glutathione (GSH) (Meister and Anderson, 1983). The glutathione system requires ATP (1) for GSH synthesis, (2) to generate NADPH as a co-factor in reduction of GSSG to GSH (Murray et al, 1993) and (3) to excrete glutathione-S conjugates with alkylating agents (Meister and Anderson, 1983).

Glutathione also indirectly regulates the activity of various ATP-dependent membrane-bound enzymes by preventing oxidation of membrane thiols that directly regulate enzyme activity, including ATPases responsible for calcium transport in mitochondria, the endoplasmic reticulum and plasma membranes (Nicotera and Orrenius, 1986). Thus, along with bcl-2, it may play a role in prevention of mitochondrial membrane damage in the presence of reactive oxygen species.

Should these protective mechanisms fail and DNA damage occur, DNA repair involves still other endergonic processes, such as the transfer of ADP-ribose from NAD to nuclear proteins by Poly (ADP-ribose) polymerase altering their structure to facilitate either DNA repair (Berger, 1985) or nuclease fragmentation and apoptosis (Yoon et al, 1996). ADP-ribose requires ATP for its synthesis. In addition, Poly (ADP-ribose) polymerase may consume large amounts of NAD (a co-factor in glycolysis) and secondarily deplete cellular ATP (Berger, 1985). Should this occur, Pedersen (1978) has reported that mitochondria can produce NADH and consume ATP in the process. The importance of ATP to DNA repair is highlighted by the fact that some have postulated that novobiocin inhibition of DNA excision repair is mediated not through inhibition of topoisomerases but through its effects on mitochondria and on lowering the ATP/ADP ratio (Downes et al, 1985). All these considerations suggest that prevention/repair of DNA damage might generate increased cellular requirements for ATP production.

In addition to damaging DNA, alkylating agents may also damage protein thiols, including a number of important membrane enzymes. Such damage up-regulates heat shock protein synthesis, another endergonic process (Liu et al, 1996). Heat shock proteins act as molecular chaperones to maintain the conformation of critical cellular proteins (Frydman and Hartl, 1996) and have been reported to co-operate with bcl-2 in antagonizing apoptosis (Strasser and Andersen, 1995).

It is possible, although statistically unlikely ($P \sim 0.006 \times 0.0002 \times 0.06 \times 0.03 \times 0.05$), that elevations of NTP, PCr, Bcl-2 protein and total GSH and significant decreases in NDP and Pi 2 h after nitrogen mustard treatment in HT-29 cells occurred purely by chance and cannot in themselves be taken as prima facie evidence for up-regulation of mitochondrial function. However, studies from other investigators also suggest that bcl-2 is involved in regulation of mitochondrial function (Lam et al, 1994; Lithgow, 1994; Magnelli et al, 1994; Myers et al, 1995; Richter et al, 1996) and have shown that the bcl-2a protein localizes to the outer mitochondrial membrane and prevents reactive oxygen species-induced calcium cycling, stabilizing the mitochondrial membrane potential and ATP levels. Smets et al (1994) showed that bcl-2 protein levels and baseline ATP levels were linearly related in six human cell lines. It has also been shown recently (Kluck et al, 1997; Yang et al, 1997) that overexpression of bcl-2 prevented efflux of holocytochrome c from mitochondria, preventing induction of apoptosis. In these systems, maintenance of ATP levels did not appear to be important during initiation of apoptosis, but post-initiation progression of apoptosis was retarded by the protonophore CCCP, suggesting a role for ATP as an energy source for this endergonic process (Richter, 1966). That ATP might be required to provide energy for apoptosis is also supported by observations that, during apoptosis not induced by mitochondrial respiratory inhibitors, mitochondrial structure and function and ATP levels are often (Murgia et al, 1992; Cossarizza et al, 1994; Mills et al, 1995) but not always (Deckwerth and Johnson, 1993) preserved until after DNA fragmentation.

In the studies of Yang et al (1997) and Kluck et al (1997), deoxyadenosine triphosphate, which might have come from DNA damage, was a co-factor with mitochondrial holocytochrome c in activation of the nuclease CPP32, suggesting a potential for cooperation between DNA damage and alterations in mitochondrial permeability to holocytochrome c in initiation of apoptosis. Our observations that ATP levels are up-regulated by bcl-2 in association with increasing levels of GSH suggest that lowering ATP levels might interfere with the process of DNA protection/repair and thereby contribute to apoptosis initiation.

The studies reported here show a temporal overlap between significant increases in NTP, PCr and bcl-2 and the initial rise in total cellular glutathione (GSH) and significant decreases in NDP and Pi at 2 h after $HN_2$ treatment in the HT-29 cell line. This suggests the possibility of bcl-2 up-regulation of ATP to support metabolic needs with DNA damage prevention/repair processes. An insignificant increase in S-phase at 2 h may also have contributed to the overall cellular ATP needs.

Bcl-2 has been reported to be up-regulated by TNF through a mechanism dependent on protein kinase C (Genestier et al, 1995) and by interleukin 2 (IL-2) and IL-7 in natural killer cells (Armant et al, 1995). Bcl-2 up-regulation has not yet been reported to occur after in vivo administration of chemotherapeutic agents (Walton et al, 1993). Tosi et al (1996) have recently observed increased bcl-2 expression after prednisolone in vitro. In these studies, we show that high-dose $HN_2$ up-regulates bcl-2 protein levels both in HT-29, a cell line that normally has low bcl-2 levels, and in MCF-7, a cell line normally expressing high levels of bcl-2. In HT-29, up-regulation of bcl-2 protein at 2 h coincides with significant increases in

© Cancer Research Campaign 1998

NTP and PCr and significant decreases in NDP and Pi, which we believe reflects presumptive evidence of up-regulation of the mitochondrial membrane potential. Nitrogen mustard-induced increases in bcl-2 may protect mitochondrial membranes from lipid oxidation from nitrogen mustard, helping to sustain the mitochondrial membrane potential. These changes in cellular energetics were associated with rising GSH levels, which suggests that they may support endergonic DNA damage prevention/repair mechanisms. Finally, as DNA damage is also a signal for apoptosis, it is attractive to speculate that bcl-2 is involved in prevention of DNA damage and DNA damage repair, as this would be in line with its other known anti-apoptosis effects (Chen and Faller, 1996).

These studies demonstrate that chemotherapy agents can up-regulate bcl-2 expression, and they link up-regulation of a specific drug resistance gene (*bcl-2*) and increased cellular GSH (which protects against nitrogen mustard damage to DNA and cell membranes) to specific $^{31}$P-MRS-observable events in vitro (increases in ATP and PCr and decreases in NDP and Pi).

### ACKNOWLEDGEMENTS

This study was supported in part by NIH Grant no. CA62184, a grant from Burroughs Wellcome and the Department of Surgical Oncology Research Fund (various donors).

### ABBREVIATIONS

MDPA, methylene diphosphonic acid; Pi, inorganic phosphate; PCr, phosphocreatine; NTP alpha, beta, gamma, nucleoside triphosphate alpha, beta, gamma phosphate resonances; ATP, adenosine triphosphate; ADP, adenosine diphosphate; $^{31}$P, phosphorus 31; MRS, magnetic resonance spectroscopy; GSH, glutathione; GSSG, glutathione disulphide; $GSH_{total}$, GSH+GSSG; EDTA, ethylene diamine tetra-acetic acid; GTP, guanosine triphosphate; CTP, cytidine triphosphate; NMR, nuclear magnetic resonance; UTP, uridine triphosphate

### REFERENCES

Ackerboom TP and Sies H (1981) Assay of glutathione, glutathione disulfide, and glutathione mixed disulfides in biological samples. *Methods Enzymol* **77**: 373–382

Armant M, Delespesse G and Sarfati M (1995) Il-2 and Il-7 but not Il-12 protect natural killer cells from death by apoptosis and up-regulate bcl-2 expression. *Immunology* **85**: 331–337

Barany M and Glonek T (1982) Phosphorus-31 nuclear magnetic resonance of contractile systems. *Methods Enzymol* **85**: 624–676

Berger NA (1985) Symposium: cellular response to DNA-damage: the role of poly (ADP-ribose) in the cellular response to DNA damage. *Radiat Res* **101**: 4–15

Berghmans K, Ruiz-Cabello J, Simpkins H, Andrews PA and Cohen JS (1992) Increase in the ATP signal after treatment with cisplatin in two different cell lines studied by $^{31}$P NMR spectroscopy. *Biochem Biophys Res Commun* **183**: 114–120

Bhaskar L, Mathan MM and Balasubramaniam KA (1995) Oxygen free radical damage during colonic ischemia/reperfusion in rats. *Mol Cell Biochem* **151**: 9–14

Burt GT, Glonek T and Barany M (1976*a*) Analysis of phosphate metabolites, the intracellular pH, and the state of adenosine triphosphate in intact muscle by phosphorus NMR. *J Biol Chem* **251**: 2584–2591

Burt CT, Glonek T and Barany M (1976*b*) Phosphorus-31 nuclear magnetic resonance detection of unexpected phosphodiesters in muscle. *Biochemistry* **15,** 4850

Chen CY and Faller DV (1996) Phosphorylation of Bcl-2 protein and association with p21Ras in ras-induced apoptosis. *J Biol Chem* **271**: 2376–2379

Cohen JS, Lyon RC, Chen C, Faustino PJ, Batist G, Shoemaker M, Rubalcaba E and Cowan KH (1987) Differences in phosphate metabolite levels in drug-sensitive and resistant human breast cancer cell lines determined by $^{31}$P magnetic resonance spectroscopy. *Cancer Res* **47**: 3396–3401

Cossarizza A, Kalashnikova G, Grassilli E, Chiapelli F, Salvioli S, Capri M, Barbieri D, Triano L, Monti D and Franceschi C (1994) Mitochondrial modifications during rat thymocyte apoptosis: a study at the single cell level. *Exp Cell Res* **214**: 323–330

Decaudin D, Gely S, Hirsch T, Castedo M, Martchetti P, Macho A, Kofler R and Kromer G (1997) Bcl-2 and bcl-x antagonize the mitochondrial dysfunction preceding nuclear apoptosis induced by chemotherapeutic agents. *Cancer Res* **57**: 62–67

Deckwerth TL and Johnson EM (1993) Temporal analysis of events associated with programmed cell death (apoptosis) of sympathetic neurons deprived of nerve growth factor. *J Cell Biol* **123**: 1207–1222

Desmoulin F, Galons JP, Canioni P, Marvaldi J and Cozzone PJ (1986) $^{31}$P nuclear magnetic resonance study of a human colon adenocarcinoma cultured cell line. *Cancer Res* **46**: 3768–3774

Downes CS, Ord MJ, Mullinger AM, Collins AR and Johnson RT (1985) Novobiocin inhibition of DNA excision repair may occur through effects on mitochondrial structure and ATP metabolism, not on repair topoisomerases. *Carcinogenesis* **6**: 1343–1352

Duke RC and Cohen JJ (1992) Morphological and biochemical assays of apoptosis. In *Current Protocols in Immunology*, Coligan JE and Kruisbeak AM (eds), pp. 3.17.1–3.17.16. John Wiley & Sons: New York

Egushi Y, Shimizu S and Tsujimoto Y (1997) Cellular ATP levels determine cell death by apoptosis or necrosis. *Cancer Res* **57**: 1835–1840

Endicott JA and Ling V (1989) The biochemistry of P-glycoprotein-mediated multidrug resistance. *Annu Rev Biochem* **58**: 137–171

Evanochko WT, Sakai TT, Ng TC, Ramakrishna N, Kim HD, Zeidler RB, Ghanta VK, Brockman RW et al (1984) NMR study of *in vivo* RIF-1 tumors. Analysis of perchloric acid extracts and identification of H-1, -31, and C-13 resonances. *Biochim Biophys Acta* **805**: 104–116

Fantini J, Galons JP, Marvaldi J, Cozzone PJ and Canioni P (1987) Growth of a human adenocarcinoma cell line (HT-29) on microcarrier beads. Metabolism studies by $^{31}$P NMR spectroscopy. *Int J Cancer* **39**: 255–260

Fough J and Trempe C (1975) New human tumor cell lines. In *Human Tumor Cell Lines in vitro*, Fough J and Trempe C (eds), pp. 115–141. Plenum: New York

Frei E, Cucchi CA, Rosowski A et al (1985) Alkylating agent resistance: *in vitro* studies with human cell lines. *Proc Natl Acad Sci USA* **82**: 2158–2162

Frydman J and Hartl F (1996) Principles of chaperone-assisted proton folding, differences between *in vitro* and *in vivo* mechanisms. *Science* **272**: 1497–1502

Genestier L, Bonnefoy-Berard N, Rouault JP, Flacher M and Revillard JP (1995) Tumor necrosis factor-alpha up regulates Bcl-2 expression and decreases calcium-dependent apoptosis in human B cell lines. *Int Immunol* **7**: 533–540

Glickson JD, Evanochko WT, Sakai TT and Ng TC (1987) *In vivo* NMR spectroscopy of tumors. In *NMR Spectroscopy of Cells and Organisms*, Gupta RK. (ed.), CRC Press: Boca Raton, FL

Haldar S, Beatty C, Tsujimoto Y and Croce C (1989) The bcl-2 gene encodes a novel G protein. *Nature* **342**: 195–198

Hemminki K and Kallama S (1986) *Reactions of Nitrogen Mustards with DNA*. IARC Scientific Publication No. **78**, pp 55–70. IARC:

Holden HT, Lichter W and Sigel MM (1973) Quantitative methods for measuring cell growth and death. In *Tissue Culture Methods and Applications*, Kruse Jr PF and Patterson Jr MK (eds), Academic Press: New York

Jevtovic-Todorovic V and Guenthner TM (1992) Depletion of a discrete nuclear glutathione pool by oxidative stress but not by butathione sulfoximine. Correlation with enhanced alkylating agent cytotoxicity to human melanoma cells *in vitro*. *Biochem Pharmacol* **44**: 1383–1393

Kaufman SH, Desnoyers S, Ottaviano Y, Davidson NE and Poirier GG (1993) Specific proteolytic cleavage of poly (ADP-ribose) polymerase: an early marker of chemotherapy-induced apoptosis. *Cancer Res* **53**: 3976–3985

Kluck RM, Bossy-Wetzel E, Green DR and Newmeyer DD (1997) The release of cytochrome c from mitochondria: a primary site for bcl-2 regulation of apoptosis. *Science* **275**: 1132–1136

Krinsky NI (1988) Membrane antioxidants. *Ann NY Acad Sci* **151**: 17–33

Lam M, Dubyak G, Chen L, Nunez G, Miesfeld RL and Diestelhorst CW (1994) Evidence that Bcl-2 represses apoptosis by regulating endoplasmic reticulum-associated $Ca^{2+}$ fluxes. *Proc Natl Acad Sci USA* **91**: 6569–6573

Laurence DJR (1962) Chain breakage of deoxyribonucleic acid following treatment with low doses of sulfa mustard. *Proc R Soc Lond* **271**: 520–530

Lithgow T, van Driel R, Bertram JF and Strasser A (1994) The protein product of the oncogene bcl-2 is a component of the nuclear envelope, the endoplasmic reticulum, and the outer mitochondrial membrane. *Cell Growth Diff* **5**: 411–417

© Cancer Research Campaign 1998

Liu H, Lightfoot R and Stevens JL (1996) Activation of heat shock factor by alkylating agents is triggered by glutathione depletion and oxidation of protein thiols. *J Biol Chem* **271**: 4805–4812

Lowry OH, Rosenbrough NJ, Farr AL and Randall RJ (1951) Protein measurement with the folin phenol reagent. *J Biol Chem* **193**: 265–275

Magnelli L, Cinelli M, Turchetti A and Chiarugi VP (1994) Bcl-2 overexpression abolishes early calcium waving preceding apoptosis in NIH-3T3 murine fibroblasts. *Biochem Biophys Res Commun* **204**: 84–90

Meister A and Anderson ME (1983) Glutathione. *Annu Rev Biochem* **52**: 711–760

Mills JC, Nelson D, Erecinska M and Pittman RN (1995) Metabolic and energetic changes during apoptosis in neural cells. *J Neurochem* **65**: 1721–1730

Mitchell P (1961) Coupling of phosphorylation to electron and hydrogen transfer by a chemo-osmotic type of mechanism. *Nature* **191**: 144–148

Murgia M, Pizzo P, Sandona D, Zanovello P, Rizzuto R and Di Virgilio F (1992) Mitochondrial DNA is not fragmented during apoptosis. *J Biol Chem* **267**: 10939–10941

Murray RK, Granner DK, Myers PA and Rodwell VW (1993) *Harper's Biochemistry*, 23rd edn. Appleton & Lange: Norwalk, CT

Myers KM, Fiskum G, Liu Y, Simmens SJ, Bredesen DE and Murphy AN (1995) Bcl-2 protects neural cells from cyanide/aglycemia-induced lipid oxidation, mitochondrial injury, and loss of viability. *J Neurochem* **65**: 2432–2240

Naruse S, Hirakawa K, Tanaka C, Higuchi T, Ueda S, Nishikawa H and Watan H (1985) Measurement of *in vivo* $^{31}P$ nuclear magnetic resonance spectra in neuroectodermal tumors for the evaluation of the effects of chemotherapy. *Cancer Res* **45**: 2429–2433

Neeman M, Eldar H, Rushkin E and Degani H (1990) Chemotherapy-induced changes in the energetics of human breast cancer cells: $^{31}P$ and $^{31}C$ NMR studies. *Biochim Biophys Acta* **1052**: 255–263

Ng TC, Evanochko WT, Hiramoto RN, Ghanta VK, Lilly MB, Lawson AJ, Corbett TH, Durant JR and Glickson JD (1982) $^{31}P$ NMR spectroscopy of *in vivo* tumors. *J Magn Reson* **49**: 271–286

Nicotera P and Orrenius S (1986) Role of thiols in protection against biological reactive intermediates. *Adv Exp Med Biol* **197**: 41–51

O'Conner PM, Jerres DK, White GA, Pines J, Hunter T, Longo DL and Kohn KW (1992) Relationship between cdc2 kinase, DNA cross linking and cell cycle perturbation induced by nitrogen mustard. *Cell Growth Diff* **3**: 43–52

Ohta K, Iwai Y, Taniguchi N, Krajewski S, Reed JC and Miyawaki T (1995) Immunoblot analysis of cellular expression of Bcl-2 family protein Bcl-2, Bax, Bcl-x, and Mcl-1, in human peripheral blood and lymphoid tissues. *Int Immunol* **7**: 1817–1825

Paris H, Terrain B, Villard V, Rousset M, Zweibaum A and Murat JC (1983) Activation of glycogen metabolizing enzymes in glucose deprived HT-29 cells. *Biochem Biophys Res Commun* **110**: 371–377

Pedersen PL (1978) Tumor mitochondria and the bioenergetics of cancer cells. *Prog Exp Tumor Res* **22**: 190–274

Pratt WB (1973) *Fundamentals of Chemotherapy*. Oxford University Press: New York

Richter C, Schweizer M, Cossarizza A and Franceschi C (1996) Hypothesis: control of apoptosis by the cellular ATP level. *FEBS Lett* **378**: 107–110

Robson CN, Lewis AD, Wolf CR, Haynes JD, Hall A, Proctor SJ, Harris AL and Hickson ID (1987) Reduced levels of drug-induced DNA cross-linking in nitrogen mustard-resistant Chinese hamster ovary cells expressing elevated glutathione D-transferase activity. *Cancer Res* **47**: 6022–6027

Sakamuro D, Eviner V, Elliot KJ, Showe L, White E and Pendergast GC (1995) c-myc induces apoptosis in epithelial cells by both p-53 dependent and p-53 independent mechanisms. *Oncogene* **11**: 2411–2118

Shidoji Y, Nakamura N, Moriwaki H and Muto Y (1997) Rapid loss in the mitochondrial membrane potential during geranylgeranoic acid-induced apoptosis. *Biochem Biophys Res Commun* **230**: 58–63

Shimizu S, Eguchi Y, Kamike W, Waguri S, Uchiyama Y, Matsuda H and Tsujimoto Y (1996) Bcl-2 blocks loss of mitochondrial membrane potential while ICE inhibitors act at a different step during inhibition of death induced by respiratory chain inhibitors. *Oncogene* **13**: 21–29

Skulachev VP (1992) Review: the laws of cell energetics. *Eur J Biochem* **208**: 203–209

Smets LA, Van den Berg J, Acton D, Top B, Van Rooji H and Verwijs-Janssen M (1994) Bcl-2 expression and mitochondrial activity in leukemic cells with different sensitivity to glucocorticoid-induced apoptosis. *Blood* **84**: 1613–1619

Smith SR, Martin PA and Edwards RHT (1991) Tumor pH and response to chemotherapy: an *in vivo* $^{31}P$ magnetic resonance spectroscopy study in non-Hodgkin's lymphoma. *Br J Radiol* **64**: 923–928

Steen RG (1989) Responses of solid tumors to chemotherapy monitored by *in vivo* $^{31}P$ nuclear magnetic resonance spectroscopy: a review. *Cancer Res* **49**: 4075–4085

Strasser A and Anderson RL (1995) Bcl-2 and thermotolerance cooperate in cell survival. *Cell Growth Diff* **6**: 799–805

Tosi P, Visani G, Ottaveoni E, Manfroi S and Tura S (1996) In vitro culture with prednisolone increases bcl-2 protein expression in adult acute lymphoblastic leukemia cells. *Am J Hematol* **51**: 261–264

Walajtys EL, Gottesman DP and Williamson JR (1974) Regulation of pyruvate dehydrogenase in rat liver mitochondria by phosphorylation–dephosphorylation. *J Biol Chem* **349**: 1857–1866

Walton MI, Whysong D, O'Connor PM, Hockenberry D, Korsmeyer SJ and Kohn KW (1993) Constitutive expression of human bcl-2 modulates nitrogen mustard and camptothecin-induced apoptosis. *Cancer Res* **53**: 1853–1861

Werhle JP, Li SJ, Rajan SS, Steen RG and Glickson JD (1987) $^{31}P$ and $^{1}H$ NMR spectroscopy of tumors *in vivo*, untreated, growth, and response to chemotherapy. *Ann NY Acad Sci* **508**: 200–215

Wilson DF, Owen C, Mela L and Weiner L (1973) Control of mitochondrial respiration by the phosphate potential. *Biochem Biophys Res Commun* **33**: 326–333

Yang J, Liu X, Bhalla K, Kim CN, Lbrado AM, Cai J, Peng T, Jones DP and Wang X (1997) Prevention of apoptosis by bcl-2: release of cytochrome c from mitochondria blocked. *Science* **275**: 1129–1132

Yao KS, Clayton M and O'Dwyer PJ (1995) Apoptosis in human adenocarcinoma HT-29 cells induced by exposure to hypoxia. *J Natl Cancer Inst* **87**: 117–122

Yoon YS, Kim JW, Kong KW, Kim YS, Choi KH and Joe CO (1996) Poly-(ADP-ribosyl)-ation of histone H1 correlates with internucleosome DNA fragmentation. *J Biol Chem* **271**: 9129–9134

Zweier JL, Jacobus WE, Korecky B and Brandejs-Barry Y (1991) Bioenergetic consequences of cardiac phosphocreatine depletion induced by creatine analog feeding. *J Biol Chem* **266**: 20296–29304

 © Cancer Research Campaign 1998